# Comprehensive immune profiling identifies alterations in adaptive and innate immune responses in granulomatosis with polyangiitis patients in remission

**DOI:** 10.3389/fimmu.2026.1726107

**Published:** 2026-03-27

**Authors:** Ming Liu, Deming Duan, Chenghao Li, Xia Hu, Jinghao Huang, Yue Tang, Steven Palayew, Lei Zhang, Christian Pagnoux, Cynthia Guidos, Jinyi Zhang, Katherine Siminovitch

**Affiliations:** 1 Mount Sinai Hospital, Lunenfeld-Tanenbaum and Toronto General Hospital Research Institutes, Toronto, ON, Canada; 2 Oujiang Laboratory (Zhejiang Lab for Regenerative Medicine, Vision and Brain Health), Wenzhou Medical University, Wenzhou, Zhejiang, China; 3 School of Life Science and Technology, State Key Laboratory of Urban Water Resource and Environment, Harbin Institute of Technology, Harbin, Heilongjiang, China; 4 College of Basic Medicine, Jinzhou Medical University, Jinzhou, Liaoning, China; 5 Vasculitis Clinic, Division of Rheumatology, Mount Sinai Hospital, University of Toronto, Toronto, ON, Canada; 6 The Hospital for Sick Children Research Institute, Program in Cell & Systems Biology, University of Toronto, Toronto, ON, Canada; 7 Department of Immunology, Faculty of Medicine, University of Toronto, Toronto, ON, Canada; 8 Department of Medicine, Faculty of Medicine, University of Toronto, Toronto, ON, Canada

**Keywords:** granulomatosis with polyangiitis, immune dysregulation, immunophenotypes, innate immunity, lymphocytes, machine learning, mass cytometry, precision medicine

## Abstract

**Objective:**

This study aimed to identify immune cell alterations in granulomatosis with polyangiitis patients in remission (rGPA) that may facilitate diagnosis and prediction of relapse.

**Methods:**

Circulating immune cells were phenotypically characterized by high-dimensional CyTOF in 59 rGPA patients and 31 healthy controls (HCs). These data together with inducible cytokine expression assays and Machine Learning (ML) methods were used to identify immunophenotypic profiles distinguishing rGPA patients from HCs and patients with higher relapse frequencies.

**Results:**

rGPA patients exhibited multiple blood cell immunophenotypic features distinct from HCs, including lymphocytopenia, a shift toward exhausted effector T cells and increased B and innate immune cell activation. Using ML methods, we identified a combination of cell features (γδ T cell depletion, monocyte and CD177^+^ neutrophil expansion, B cell depletion) distinguishing rGPA patients from HCs and cytokine expression profiles among patients (increased IL-8 in monocytes, decreased IL-10 in monocytes and cDC2 cells) associated with relapse frequency. Two ML-based risk scores were developed and respectively shown to accurately discriminate rGPA cases from HCs and rGPA patients with more frequent disease relapse.

**Conclusions:**

Our findings reveal distinct patterns of immune dysregulation in rGPA patients and demonstrate potential for ML methods to facilitate disease diagnosis and outcome prediction based on immunophenotypic data.

## Introduction

Granulomatosis with polyangiitis (GPA) is a severe chronic autoimmune disease characterized by relapsing necrotizing vasculitis of small blood vessels and predominantly involving the upper and/or lower respiratory tract and kidneys (1–3). Advances in immunosuppressive therapy have dramatically improved survival in GPA, but disease relapse after successful treatment remains a significant source of high morbidity (4–7). Serologic and cellular candidate markers for detecting disease activity in GPA have been reported, but reliable biomarker(s) for predicting disease relapse are lacking. The development of such biomarker(s) and consequent opportunity to mitigate likelihood of recurrence would address a major unmet need in the management of GPA (8–11).

Multiple immune cell aberrancies have been identified in GPA and other ANCA-associated vasculitis (AAV), with some alterations correlating with disease activity and relapse risk (12–14). These changes include association of active disease with reduction of regulatory T cell number and function and altered CD4^+^ effector T, T follicular helper (Tfh) and T helper (Th) 17 cell frequencies (13–17). Alterations in frequency and/or function of selected B lineage subsets, such as regulatory B cells and plasmablasts, and in the numbers and/or activation of monocytes, neutrophils and natural killer (NK) cells have also been observed in patients with active disease and implicated in disease pathophysiology and/or progression (18–28).

While the collective data establish the association of GPA with profound dysregulation of immune responses, most such studies have primarily focused on a few or single immune cell lineages. As such, data on the amalgam of immune cell alterations associated with GPA and critical clinical outcomes, such as relapse, are limited and differ across studies (29). Thus, identifying a composite of cell alterations associated with key GPA outcomes requires broader immune cell profiling.

In this study, we have investigated the immune landscape in rGPA patients to identify immunophenotypic features in peripheral blood that distinguish these patients from HCs and may be associated with increased likelihood of disease relapse. To enable comprehensive characterization of circulating immune cell populations, we employed CyTOF, a high-dimensional single cell technology platform used successfully to elucidate immunologic features of other autoimmune diseases (30–32). By combining this approach with machine-learning methods, we have identified multiple features of circulating immune cells that distinguish rGPA patients from HCs and that may aid in identifying likelihood of relapse of disease.

## Materials and methods

### Study design and participants

Fifty-nine patients diagnosed with GPA according to the 1990 American College of Rheumatology classification criteria and 31 age- and sex-matched HCs were recruited from the Rheumatology Centre at Mount Sinai Hospital between January 2017 and May 2020. All enrolled patients were between 32 and 66 years of age and were in clinical remission at the time of inclusion, as defined by a Birmingham Vasculitis Activity Score (BVAS) for Wegener’s Granulomatosis of 0 (32–34). Age, sex, disease duration, organ involvement, history of relapse, ANCA positivity and disease remission status, BVAS score and medications at the time of sampling were recorded. The study was approved by the Mount Sinai Hospital Research Ethics Board and informed consent obtained from all patients or their legal representatives.

### Blood sampling and processing and mass cytometry staining and acquisition

A schematic showing study design and analytical pipeline is provided in **Supplementary Figure S1**. Details on blood processing are provided in **Supplementary Materials and Methods**. Briefly, peripheral venous whole blood from rGPA patients and HCs was collected and immediately processed. PBMCs were obtained by density gradient centrifugation and left untreated or stimulated with PMA (50 ng/ml) and ionomycin (500 ng/ml) or LPS (1 μg/ml). Cells were then stained with immunophenotyping antibody panels (**Supplementary Table S1**) and subjected to CyTOF analysis.

### Data preprocessing and clustering

FCS files from all patients and HCs were imported into R (version 4.4.2). Channel names were standardized, and events with zero values or missing data removed. Debris and doublets were excluded by gating on FSC-A (>20,000) and SSC-A (>10,000). Each file was randomly downsampled to 2,000 cells (Flow Core) and merged into a single dataset. Batch effects were corrected using the Harmony package (version 1.2.3). Manual gating was performed in Cytobank using lineage-defining markers. An arcsinh transformation was applied to the 25 surface markers (PBMC panel; cytokine and activation markers were excluded). A 10×10 FlowSOM self-organizing map was generated, followed by meta-clustering using ConsensusClusterPlus (k = 27, 100 repetitions, pItem = 0.8). viSNE was performed on the transformed data (dims = 2, θ = 0.5, seed = 123). Meta-clusters were annotated based on median marker expression relative to predefined thresholds and plots generated using ggplot2.

### Unsupervised hierarchical clustering

Immune cell features with significantly different proportions between rGPA patients and HCs were identified using two-sided Mann–Whitney U test (*p* < 0.05). To ensure biological relevance, only immune features with a median frequency greater than 1% or a mean metal intensity (MMI) greater than 1 across all samples were retained. Missing marker values were imputed using the within-sample median to preserve data completeness and minimize bias. Unsupervised hierarchical clustering was performed in R using the K-means algorithm, focusing only on the selected immune features. Based on dendrogram structure, samples were divided into three clusters using the cutree function. For visualization, immune features were Z-score normalized by row, and a heatmap generated using the ComplexHeatmap package (version 2.18.0). Sample annotation (such as age and relapse status were added using HeatmapAnnotation, and heatmap columns split based on the derived cluster labels.

### Construction of machine learning models

To identify immune cell biomarkers associated with disease risk and relapse, we implemented a machine learning approach that combines random forest classification with Recursive Feature Elimination; features selection and model training were conducted in R using the caret package (version 6.0-94). Two predictive models were developed: (1) Multi-Immune Cell Feature GPA Risk Probability Model (MIGRPscore), which distinguishes patients with rGPA from HCs; (2) Multi-Immune Cell Feature GPA Relapse Risk Probability Model (MIGRRPscore), which predicts relapse risk based on three selected subsets. Details of machine learning construction are provided in **Supplementary Materials and Methods**.

### Statistical analysis

All identified immune cell subsets were statistically analyzed using SPSS (version 26.0; IBM Corporation). Quantitative data are presented as mean ± standard deviation for bar graphs, and categorical variables as percentages and MMI. All *P*-values were calculated using two-side Mann-Whitney U test and were corrected for multiple comparisons using the Benjamini-Hochberg adjustment at 5%. In each boxplot, the center line indicates the median; box limits indicate the first and third quartiles; whiskers extend to 1.5× the interquartile range; all outliers beyond the whisker range are displayed as points. *P*-values were calculated using a two-tailed paired Wilcoxon test.

## Results

### Characteristics of rGPA patients at time of study entry

Clinical and demographic characteristics of the enrolled rGPA patients are provided in **Table 1**. Patients mean age was 49.2 ± 17 years and 57.6% were female. Among HCs, mean age was 50.7 ± 14 years and 58.1% were female. At study entry, 37.3% of patients had no prior history of relapse following initial treatment, 25.4% had one relapse, 37.3% had more than one relapse and 88.1% of patients were receiving at least one immunomodulatory medication.

**Table 1:** Patient characteristics at study inclusion.

Characteristic	rGPA (n=59)
Age, means±SD, y	49.2±17
Sex, n (%)	
male	25 (42.4)
female	34 (57.6)
Relapsed	
0	22 (37.3)
1	15 (25.4)
2	11 (18.6)
3	6 (10.2)
4	5 (8.5)
Last time relapsed, y	
≤1	10 (16.9)
1-3	14 (23.7)
≥3	13 (22.0)
Progression, y	
≤1	6 (10.2)
1-3	18 (30.5)
3-6	15 (25.4)
6-9	8 (13.5)
>9	12 (20.3)
Treatment Status at the Time of Blood Collection	
Prednisone	33 (55.9)
Rituximab	21 (35.6)
Azathioprine	17 (28.8)
Cyclophosphamide	5 (8.5)
Methotrexate	3 (5.1)
None	7 (11.9)
Organ Involvement at initial disease	
Ear, nose, and throat	52 (88.1)
Pulmonary	43 (72.88)
Renal	32 (54.2)
Arthritis/ Anthralgia	28 (47.4)
Nodules	22 (37.3)
Nervous system	16 (27.1)
ANCA-positive at diagnosis, n(%)	
Anti-PR3	53 (89.8)
Anti-MPO	5 (8.5)
ANCA-positive at inclusion, n(%)	
Anti-PR3	24 (40.7)
Anti-MPO	5 (8.5)

Data are shown for remission granulomatosis with polyangiitis (GPA; n = 59).Age is presented as mean ± SD; all other variables are presented as n (%). "Relapsed" indicates the number of prior relapses. "Last time relapsed" denotes the interval (years) from the most recent relapse to inclusion. "Progression" denotesthe disease duration of patients from time of diagnosis. Treatment status refers to ongoing therapies at the time of blood collection. The "None" refers to patients who were in clinical remission who were not receiving any immunosuppressive therapy at the time of blood sampling. Organ involvement reflects manifestations at initial disease presentation. ANCA status (anti- PR3 and anti-MPO) is reported at diagnosis and at inclusion.

### Immunophenotypic differences between patients and controls

**Figure 1:**
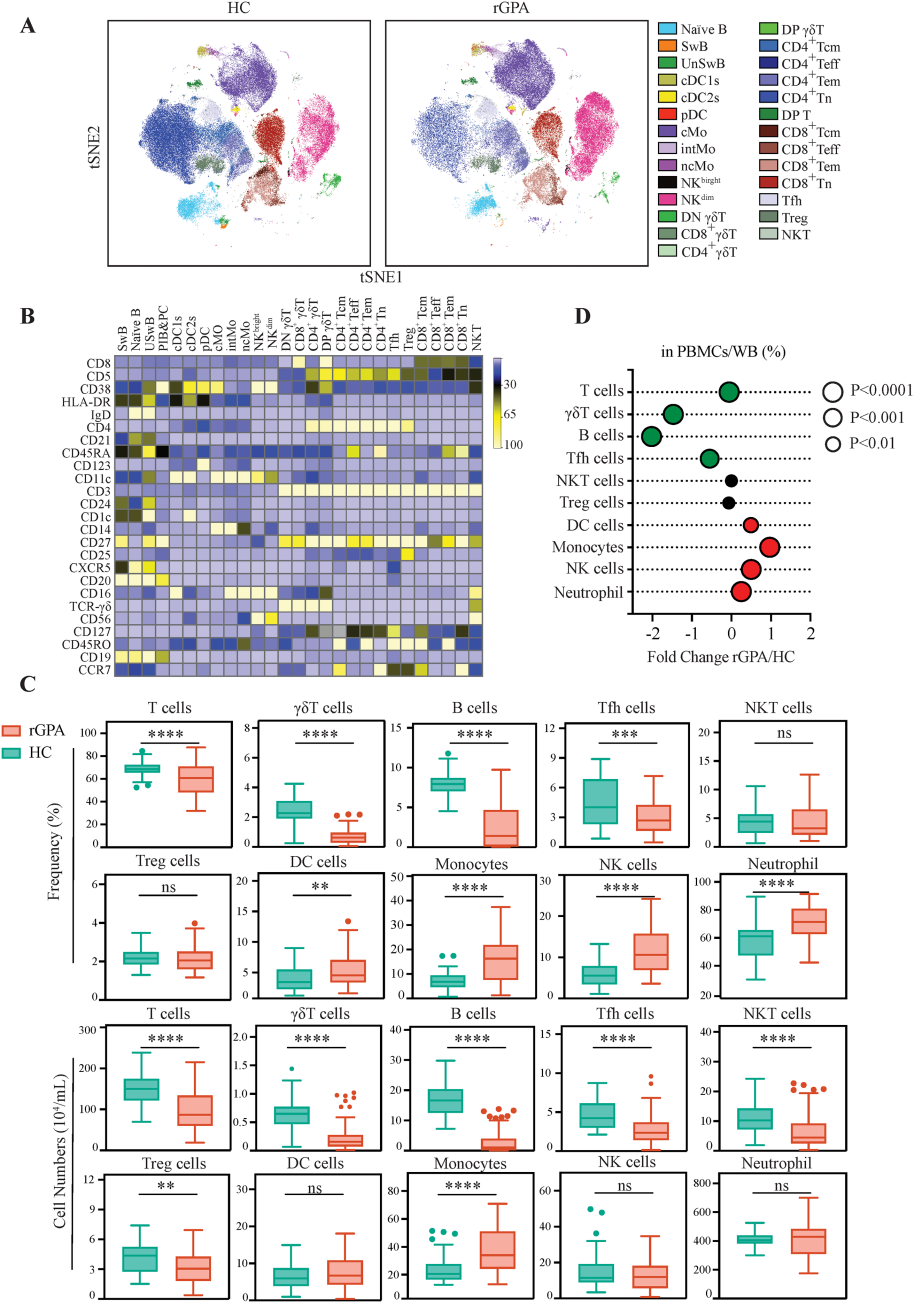
Comprehensive Immunophenotyping Unveils Distinct Immune Alterations in rGPA Patients. HCs and rGPA patient PBMCs were stained and analyzed via mass cytometry. For each sample, 100,000 events were recorded, and data analysis conducted using Cytobank. **(A)** viSNE plots of 180,000 single cells (HCs, n=31; rGPA, n=59; 2,000/sample) color coded by merged clusters and stratified by group. **(B)** Heatmap showing normalized mean metal intensities (27 clusters, 25 markers). **(C)** Frequencies (PBMCs/WB) and cell counts of adoptive and innate subsets, including T, γδT, B, Tfh, NKT, Treg, DCS, NK cells, monocytes in PBMCs, and neutrophils from WB, were compared between HCs and rGPA patients. **(D)** Bubble chart showing the fold change and significance; size indicates statistical significance: black-no significant, green-decreased, red-increased in rGPA. N=31 for HCs and 59 for rGPA samples. Data are presented as Tukey-style box-and-whisker plots. Center line: median; box: first/third quartiles; whiskers: 1.5xIQR; outliers shown. All P-values were calculated using Mann-Whitney U test and were corrected for multiple comparisons using the Benjamini-Hochberg adjustment at 5%. **P<0.01, ***P<0.001, ****P<0.0001; ns: not significant.

WB and PBMC samples from 59 rGPA patients and 31 HCs were assessed by CyTOF. For computational efficiency and comparing density across samples, viSNE was used to visualize cellular heterogeneity and identified clusters then manually annotated (**Supplementary Figure S3**) as shown in **Figure 1A**. Expression of key phenotypic markers across cell populations is shown as a heat map in **Figure 1B**. Based on marker expression profiles from non-downsampled datasets, six major immune cell lineages were identified: T and B lymphocytes, dendritic cells (DCs), monocytes, natural killer (NK) cells and neutrophils (**Supplementary Figure S2**). Cell population proportions at the sample level are summarized in **Figure 1D**. Specifically, relative frequencies of T cells (59.99% *vs* 68.75%, *p* < 0.0001) and B cells (1.46% *vs* 7.69%, *p* < 0.0001) as well as absolute lymphocyte numbers were significantly lower (*p* < 0.0001) in rGPA patients compared with HCs (**Figure 1C**). In contrast, relative frequencies of DCs (4.70% in rGPA *vs*. 3.33% in HCs, *p* = 0.0016), monocytes (15.69% *vs* 7.16%, *p* < 0.0001), NK cells (10.42% *vs* 5.83%, *p* < 0.0001) and neutrophils (73.88% *v*. 61.56%, *p* < 0.0001) were significantly higher in patients, although only monocytes showed increased absolute numbers in patients versus controls.

### Adaptive immune dysregulation in rGPA patients

**Figure 2:**
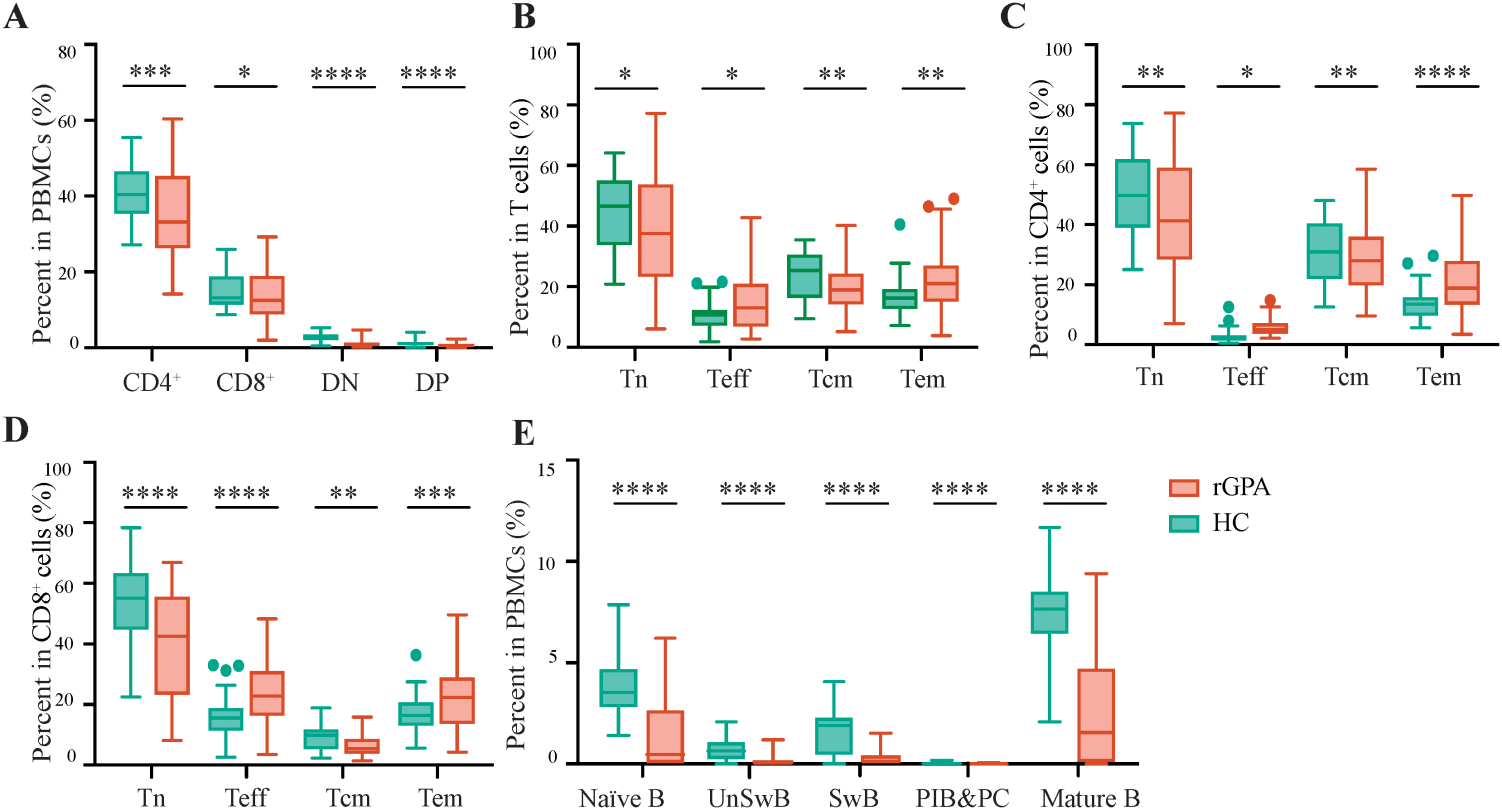
T and B lymphocyte frequencies are decreased in rGPA patients. **(A)** Frequencies of CD4^+^, CD8^+^, CD4^-^CD8^-^ (DN) and CD4^+^CD8^+^ (DP) T cells in PBMCs from HCs and rGPA patients. **(B, D)** Frequencies of naïve (Tn), effector (Teff), central memory (Tcm) and effector memory (Tem) T cells in total T **(B)**, CD4^+^T **(C)** and CD8^+^T **(D)** cells in PBMCs from HCs and rGPA patients. **(E)** Frequencies of naïve, non-class-switched memory B (UnSwB) class-switched memory (SwB), mature B cells and plasmablasts and plasma (PIB&PC) in PBMCs from HCs and rGPA patients. N=31 for HCs and 59 for rGPA samples. Data are presented as Tukey-style box-and-whisker plots. Center line: median; box: first/third quartiles; whiskers: 1.5xIQR; outliers shown. Two sided Mann-Whitney U test was used to determine P values, and false discovery rate (FDR) was controlled at 5% using the Benjamini-Hochberg procedure. *P<0.05, **P<;0.01, ***P<0.001, ****p<0.0001; ns: not significant.

Considering the critical roles for T and B lymphocytes in GPA pathogenesis, a gating approach and additional markers were used to characterize major lymphocyte subsets and their activation status (**Supplementary Figure S2**; **Supplementary Table S3**) (35, 36). This analysis revealed significantly lower absolute numbers of γδ T (*p* < 0.0001), follicular helper T (Tfh; *p* < 0.0001), regulatory T (Treg; *p* = 0.0023) and natural killer T (NKT; *p* < 0.0001) cells in patients compared with HCs, although altered frequencies were observed in only γδ T cells (0.70% in rGPA *vs.* 2.30% in HCs, *p* < 0.0001) and Tfh cells (2.88% in rGPA *vs.* 4.37% in HCs, *p* < 0.0001) (**Figures 1C, D**). CD4^+^, CD8^+^, CD4^+^CD8^+^, and CD4^-^CD8^-^ T cell frequencies were also significantly reduced in patients compared with HCs (*p* = 0.0007, *p* = 0.0320, *p* < 0.0001, *p* < 0.0001, respectively), consistent with broad T cell diminution (**Figure 2A**). Among T cells, CD4^-^CD8^-^ and CD4^+^CD8^+^ T cells were the most significantly reduced subsets, with mean decreases of 1.89-fold and 2.33-fold, respectively, relative to HCs (**Figure 2A**; **Supplementary Figure S4**). CD4^+^ and CD8^+^ single-positive T cell frequencies were more modestly reduced (about 1.10-fold). Importantly, further stratification of the CD4^+^ and CD8^+^ T cell compartments revealed major shifts in subset distribution, with effector and effector memory populations (defined by CCR7, CD45RA and CD45RO profiles) being significantly expanded (about 1.31 fold) and naïve and central memory subsets reduced by more than 1.20-fold in patients compared with HCs (**Figures 2B–D****;** **Supplementary Figure S4**).

Consistent with previous reports showing decreased frequency of circulating γδ T cells in GPA patients (37), the reduced γδ T cell frequencies apparent in rGPA patients was primarily driven by a marked decline in Vδ2^+^ γδ T cells which comprised 83.79% of the total γδ T cell population in patients and 96.58% in HCs (*p* = 0.0023) (**Supplementary Figures S5A, B**). Patient γδT cells also showed lower expression of CD56 and increased expression of PD-1, an exhaustion-like phenotype suggestive of diminished cytotoxic potential and persistent immune dysregulation (**Supplementary Figures S5C, D**). The frequencies of IFNγ, CCL4, and TNF-α producing Vδ2^+^ cells did not differ between patients and HCs (**Supplementary Figure S5E**).

B cell number and frequency were also markedly reduced in rGPA patients (**Figure 1C**), with the mean frequencies of naïve (CD3^-^CD19^+^IgD^+^CD27^-^; 1.89% *vs* 3.80%), non-class-switched memory (CD3^-^CD19^+^IgD^+^CD27^+^; 0.16% *vs* 0.71%), class-switched memory (CD3^-^CD19^+^IgD^-^CD27^+^; 0.29% *vs* 1.56%), mature (CD3^-^CD19^+^CD38^-/+^; 2.76% *vs* 7.18%) B cells and plasmablasts (CD3^-^CD19^+^IgD^-^CD27^+^CD38^++^; 0.01% *vs* 0.03%) all reduced by >2-fold (all *p* < 0.0001) in patients compared with HCs (**Figure 2E**).

### Expansion of innate immune cell subsets in rGPA

**Figure 3:**
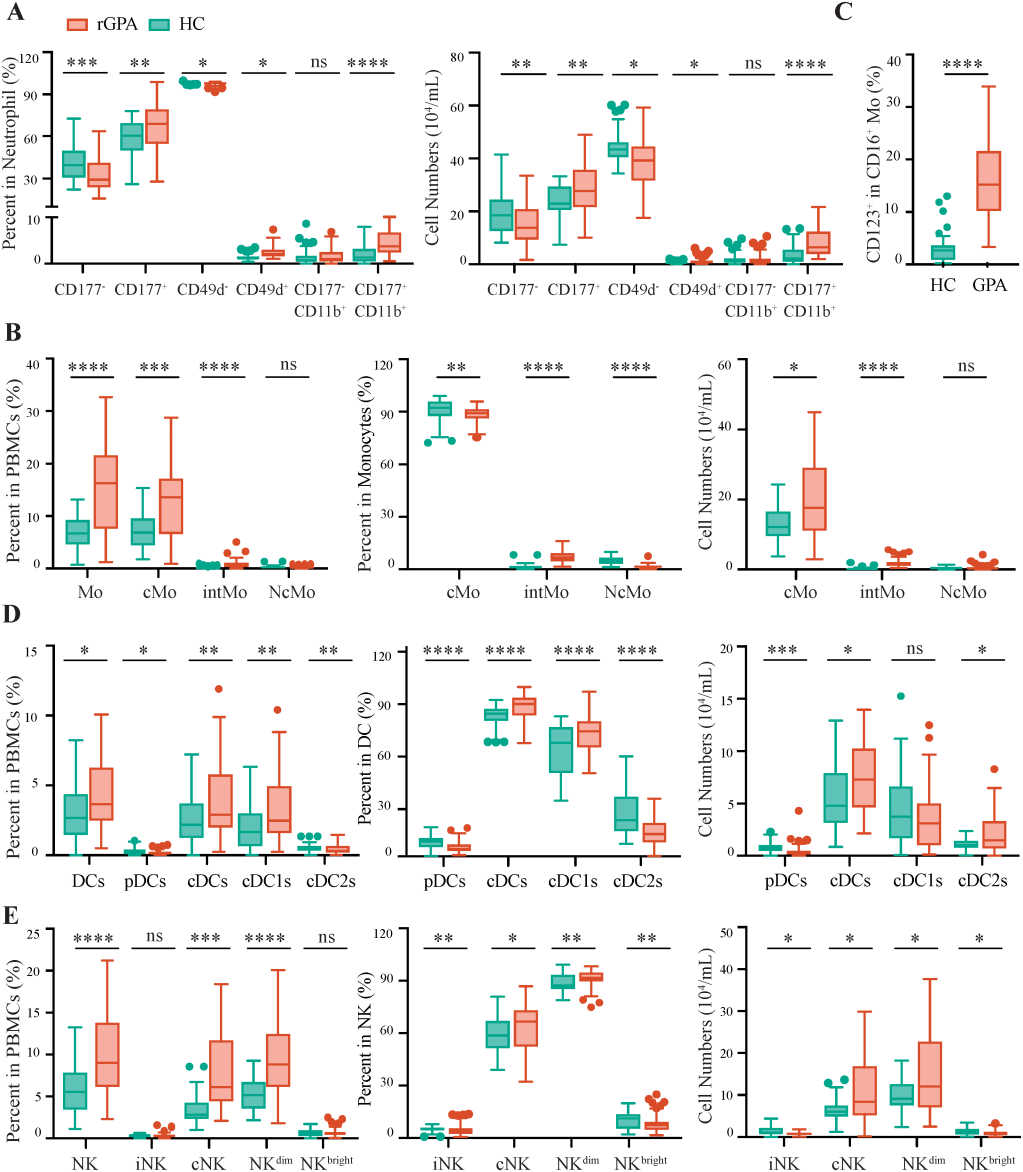
Expansion of Innate Immune Cell Populations in rGPA Patients. **(A)** Frequencies (left) and counts (right) of neutrophil subsets in whole blood from HCs and rGPA patients:: CD177^-^/CD177^-^, CD49d^-^/CD49d^+^, CD177^-^ CD11b^+^/CD177^+^CD11b^+^. **(B)** Frequencies of monocytes and monocyte subsets in PBMCs (left) and monocyte subsets in total monocytes (middle) and of monocyte subset cell numbers (right) in patients and controls. **(C)** Frequencies of CD123^+^ cells within CD16^+^ monocyte population from HCs and rGPA patients. **(D)** Frequencies of DCs and DC subsets in PBMCs (left) and DC subsets in total DCs (middle), and subset cell numbers per ml (right). **(E)** Frequencies of NKs and NK cell subsets in PBMCs (left) and NK cell subsets in total NKs (middle), and subset cell numbers (right). N=31 for HCs and 59 for rGPA samples. Data are presented as Tukey-style box-and-whisker plots. Center line: median; box: first/third quartiles; whiskers: 1.5xIQR; outliers shown. Two-sided Mann Whitney U test was used to determine P values, and false discovery rate (FDR) was controlled at 5% using the Benjamini-Hochberg procedure.*P<0.05, **P<0.01, ***P<0.001, ****P<0.0001; ns: not significant.

Because frequencies of innate immune cell populations were altered in rGPA patients, the phenotypic properties of these populations were further explored. Both frequency and absolute numbers of neutrophils expressing CD177, a glycoprotein receptor that facilitates membrane expression of PR3 (38, 39), were increased in patients (67.63% of total neutrophils *vs* 59.76% in HCs; *p* = 0.0022). A CD11b^+^CD177^+^ neutrophil subset was also expanded in patients (*p* < 0.0001), suggesting an increased frequency of activated cells in the neutrophil pool. Consistently, expression of CD49d, an adhesion molecule involved in neutrophil trafficking, was significantly increased in rGPA versus HC neutrophils (*p* = 0.010) (**Figure 3A**). Frequencies of neutrophils expressing CD18, CD35 and CD63 were, however, comparable in patients and HCs (data not shown), suggesting selective expansion of activated CD177^+^ neutrophils among patients.

Cytometric analyses also revealed number of monocytes, another leukocyte population with key roles in inflammation (26), to be increased from a mean of 2.1 × 10^5^ cells/mL in HCs to 3.4 × 10^5^ cells/mL in patients (*p* < 0.0001) (**Figures 1C**, **3B**). This alteration was associated with expansion of classical (CD14^++^CD16^-^; *p* = 0.0002) and intermediate stage (CD14^++^CD16^+^; *p* < 0.0001), but not non-classical (CD14^+^CD16^+^) monocyte frequencies. A subset of monocytes co-expressing CD16 and the IL3 receptor alpha chain, CD123, was also markedly expanded in rGPA patients (16.55% *vs* 3.14% in HCs, *p* < 0.0001) (**Figure 3C**). CD123 is typically expressed in plasmacytoid DCs (pDCs), but monocyte expression of this receptor has been reported in other autoimmune diseases and interpreted as distinguishing an important pro-inflammatory monocyte subset (40).

The relative increases in DC and NK cell frequencies observed in rGPA patients were also further explored (**Figure 1C**). Within the DC pool, increased cell frequency was driven by expansion of type 1 conventional DCs (cDC1s; 69.99% of total DCs in patients *vs* 60.10% in HCs; *p* < 0.0001) (41). In contrast, compared with HCs, patients displayed lower frequencies of cDC2s (13.31% *vs* 22.72%, respectively; *p* < 0.0001) and pDCs (5.08% *vs* 8.22%; *p* < 0.0001) (**Figure 3D**).

NK cell subpopulations were also explored using the CD56^dim^CD16^+^ and CD56^bright^CD16^-^ phenotypes to define cytotoxic/mature and immature NK cells, respectively (42, 43). This analysis revealed CD56^dim^ cells to be enriched (91.14% *vs* 88.52%; *p* = 0.0012) and CD56^bright^ NK cell frequencies and counts significantly reduced (*p* = 0.0071 *vs p* = 0.0120) among the NK population in patients compared to controls. Consistently, frequencies of cytotoxic/mature NK cells were increased (63.06% *vs* 58.80%, *p* = 0.018) and immature NK cells reduced (4.47% *vs* 5.12%) in patients compared to HCs (p=0.001) (**Figure 3E**). These findings reveal skewing of both DC and NK compartments toward terminal differentiation in rGPA patients, consistent with a generalized expansion and activation of innate immune cell subpopulations despite patient clinical remission status.

### Altered inflammatory cytokine expression in adoptive and innate immune cells

**Figure 4:**
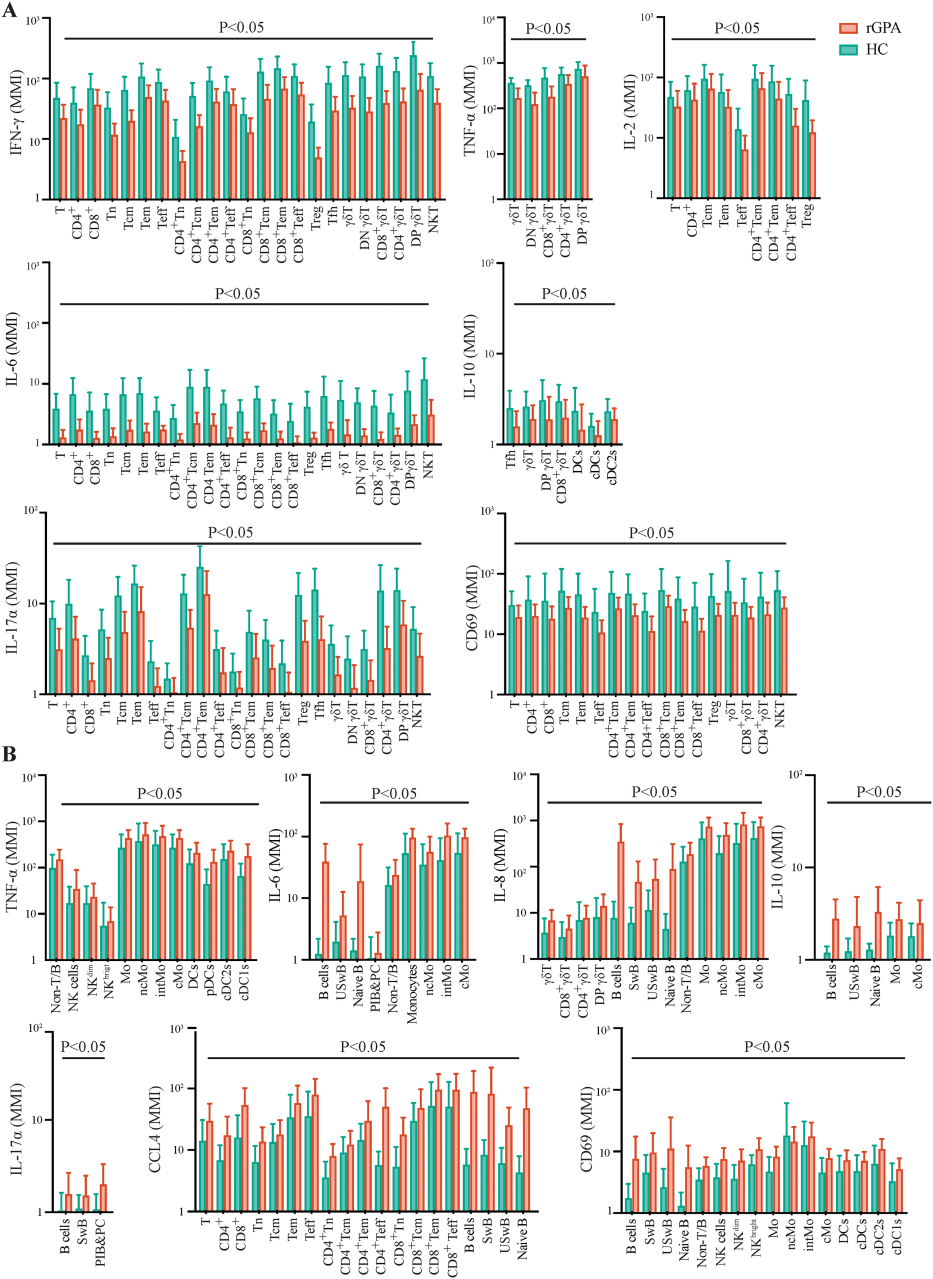
Cytokine expression levels in adaptive and innate cell subsets in PBMCs from rGPA patients and HCs. **(A)** Examples of cytokines showing lower expression in rGPA versus HCS cells after PMA/lonomycin- or LPS-stimulation: IFN-γ, TNF-α, IL-2, IL-6, IL-10, IL-17α, and CD69. **(B)** Examples of cytokines showing higher expression in rGPA versus HC cells following PMA/lonomycin- or LPS-stimulation: TNF-α, IL-6, IL-8, IL-10, IL-17α, CD69, and CCL4. Data are from 31 HCs and 59 rGPA patients and presented as mean ± SD. Statistical significance was assessed using two-sided Mann-Whitney U test and were corrected for multiple comparisons using thr Benjamini-Hochberg adjustment at 5%.

To explore the impact of the cellular alterations observed in rGPA patients on cell cytokine production, levels of cytokine expression induced by PMA/ionomycin or LPS were compared between rGPA patient and HC blood cells. This analysis revealed a complex composite of differences in inducible cytokine expression between cells from patients and HCs (**Figure 4**; **Supplementary Figure S6**). For example, a significant decrease in IL-2 production was observed in patient total T and CD4^+^ T cells and in total T and CD4^+^ central memory, effector memory and effector T cells and Tregs (all *p* < 0.05). However, IL-2 expression levels in CD8^+^ T cells and their subsets, Tfh cells, γδ T cells, NKT cells, B cell subsets, NK cells, monocytes, and DCs were not significantly different in patients versus HCs. By contrast, IL-6 expression was significantly lower (*p* < 0.05) in total T and all CD4^+^ and CD8^+^ subsets (naïve, central memory, effector memory, and effector populations), Tregs, Tfh, γδ T, CD4^-^CD8^-^ and CD4^+^CD8^+^ T cells, and NKT cells of patients compared with HCs. Additionally, TNF-α expression was significantly reduced (*p* < 0.05) in γδ T cells in patients compared with HCs, but levels in all other T cell subsets were comparable in the two groups. IL-10 expression was markedly diminished in Tfh cells and γδ T cells from rGPA patients compared with HCs (all *p* < 0.05), but not significantly different between the two groups in other T cell subsets. Expression of IL-17*α*, IFN-γ, and CD69, a marker of recent cell activation, were also consistently lower (all *p* < 0.05) across nearly all T cell subsets, and NKT cells from patients compared with HCs.

In contrast, cytokine expression levels in B and myeloid cells from rGPA patients were generally consistent with increased cell activation (**Figure 4**; **Supplementary Figure S6**). IL-6 expression, for example, was significantly upregulated (*p* < 0.05) in patient compared to control naïve B and non-class-switched memory B cells and all monocyte subsets studied. TNF-α expression was no different between patient and control B cells, but was higher (*p* < 0.05) in patient NK cell, monocyte and DC populations. Patient monocyte and B cell populations also showed significantly elevated (*p* < 0.05) levels of IL-8, while IL-10 expression was increased (*p* < 0.05) in patient monocytes, but decreased across all DC subsets. CD69 expression was higher (*p* < 0.05) in patient B cells, most B cell subsets, NK cells, monocytes and some DC subtypes compared with HCs. CCL4 and IL-17α levels were both significantly elevated (*p* < 0.05) across selective B cell subpopulations from rGPA patients, with IL-17α also relatively increased in plasmablasts. CCL4 expression levels in NKT, NK cells, monocytes, or DC cells did not differ in patients versus controls.

Together, these findings reveal a dichotomous immune landscape in rGPA patients, characterized by suppressed cytokine production and activation marker expression among T cells, contrasted with heightened proinflammatory responses and activation signatures in B and myeloid cell compartments.

### Immune cell profiling reveals GPA disease-associated and relapse-associated signatures

**Figure 5:**
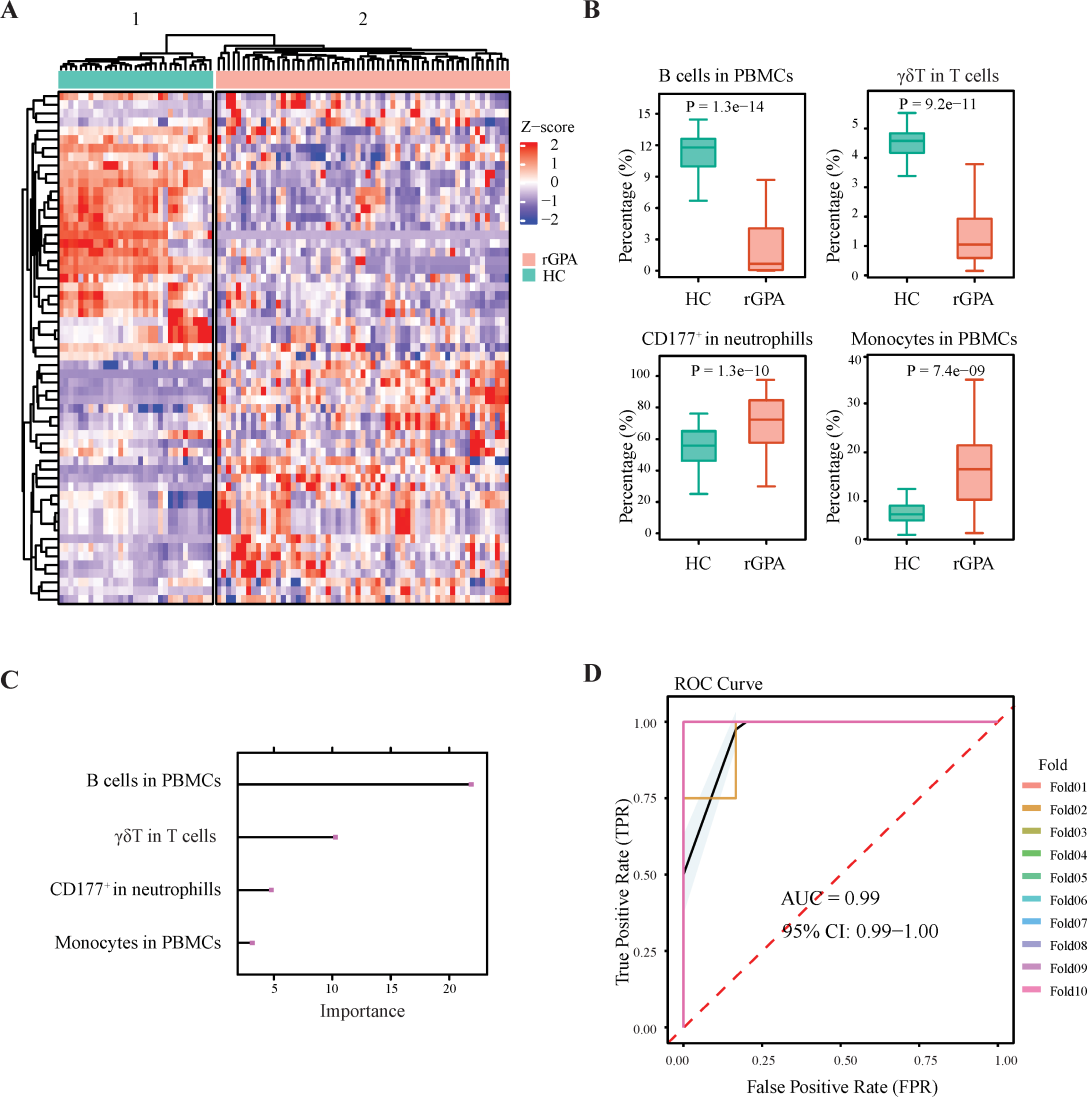
ML methods facilitate immune profiling-based distinction of rGPA patients from healthy controls. **(A)** Heatmap showing unsupervised hierarchical clustering of immune cell subsets with significantly different proportions between rGPA (n=59) and HCs (n=31). Rows represent subjects and columns show immune cell frequencies. **(B)** Boxplots of four immune biomarkers identified by RFE. Center line: median; box: first/third quartiles; whiskers: 1.5xIQR; outliers shown. P-values by two-tailed paired Wilcoxon test. **(C)** Diagnostic performance of the MIGRPscore by ten-fold cross-validation. Colored lines: receiver operating characteristic curves (ROC) of validation folds; black line: mean ROC; blue shading: 95% CI. **(D)** LASSO-based feature importance of the four biomarkers; bar color intensity indicates normalized importance (0-1).

Because the rGPA patients and HCs studied differ with respect to multiple immunophenotypic features, we applied machine learning methods to identify combinations of cell features that best differentiate rGPA patients from HCs. Results of initial Wilcoxon rank-sum test identified 59 features significantly different between the two groups, including 30 upregulated and 29 downregulated cell subset frequencies in patients compared with HCs (**Supplementary Figure S7A**). Using unsupervised hierarchal clustering, sets of marker expression patterns distinguishing patients from HCs were identified (**Figure 5A**) and then refined using the recursive feature elimination (RFE) algorithm with 10-fold cross-validation repeated 5 times. From this analysis, four key immune cell features emerged as candidate biomarkers for distinguishing patients from HCs, including γδT cell frequency among total T cells, CD177 expression in neutrophils and monocyte and B cell frequencies in PBMCs (**Figure 5B, C**). By training a random forest classifier on the four key immune biomarkers, we developed a multi-immune cell feature GPA risk probability score (**Supplementary Figure S7B**) shown by receiver operating characteristic (ROC) analysis to demonstrate strong predictive performance for disease status, with an area under the curve (AUC) of 0.99 (95% CI: 0.99–1.00) in cross-validation analysis (**Figure 5D**).

**Figure 6:**
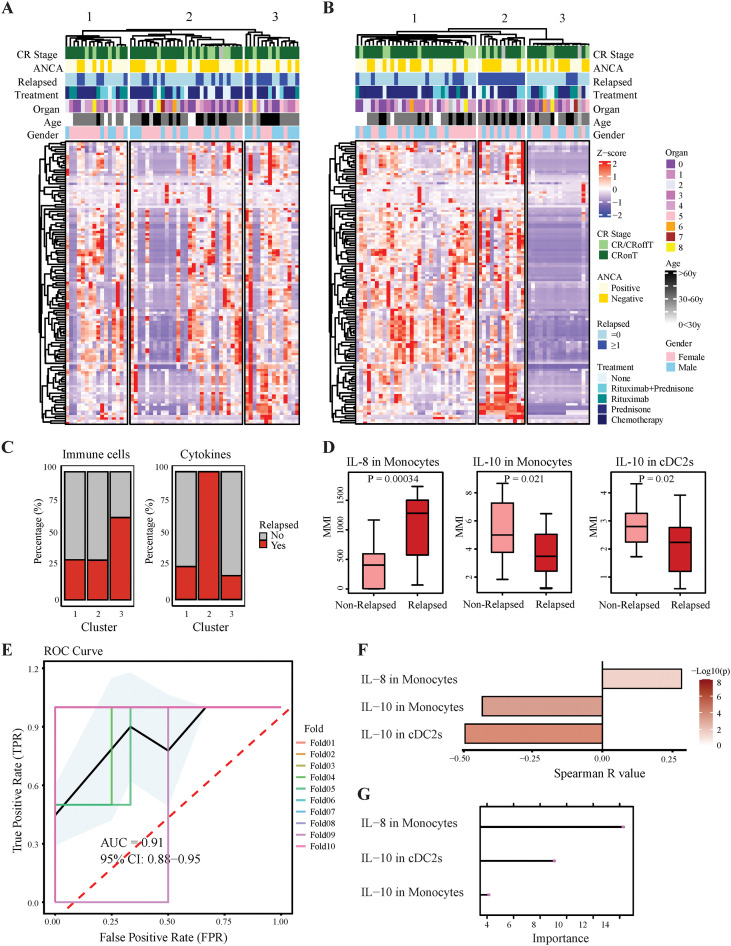
ML-based identification of relapse-associated immune profiles in rGPA patients. **(A)** Heatmap showing unsupervised hierarchical clustering of immune cell frequencies between HCs and rGPA. **(B)** Heatmap showing cytokine expression across immune cell populations, indicated as row-based log z-scores. **(C)** Clustering of immune cell frequencies (left) and cytokine expression (right) in rGPA, each yielding three clusters; bar charts show relapse frequencies. **(D)** Boxplots of three immune biomarkers identified via RFE, comparing relapsed and non-relapsed patients. **(E)** MIGRRPScore performance by ten-fold cross-validation; colored lines: ROC curves per fold; black: mean ROC; blue shading: 95% CI. **(F)** Spearman correlations between biomarker proportions and relapse frequency; color intensity reflects p-value. **(G)** LASSO-based feature importance of the biomarkers; bar color intensity: normalized importance (0-1).

Unsupervised hierarchal clustering based on cytokine expression profiles was also used to explore the immunophenotypic differences among rGPA patients. Results suggested the existence of three patient clusters, but no differences were detected among these groups with regards to key clinical features, including ANCA titers, relapse frequency, treatment regimen, organ involvement, age, sex or complete remission (**Figure 6A**). However, among three patient clusters predicated on cytokine expression profiles (**Figure 6B**), two (clusters 1 and 3) were characterized by lower cytokine expression levels and a prominence of patients with a history of low frequency disease relapse, while the third (cluster 2) was enriched for patients with high cytokine expression levels and a history of multiple relapses (**Figure 6C**). By applying the RFE algorithm (with 10-fold cross-validation repeated 5 times), we identified three distinct immune cell cytokine expression features significantly associated with increased frequency of disease relapse: increased IL-8 in monocytes and decreased IL-10 in monocytes and cDC2s cells (**Figure 6D**; **Supplementary Figure S7C**). ROC analysis of a multi-immune cell feature GPA relapse risk probability (MIGRRP) score developed from these three features revealed an AUC of 0.91 (95% CI: 0.88–0.95) (**Figure 6E**). Spearman correlation analysis, performed to assess the strength and direction of the relationships between these three features and relapse frequency, revealed statistically significant associations with relapse frequency (**Figure 6F**). Specifically, IL-8 in monocytes was positively correlated with relapse frequency (Spearman’s *r* = 0.28, *p* = 0.0307), whereas IL-10 in cDC2s (Spearman’s *r* = -0.49, *p* = 0.0001) and IL-10 in monocytes (Spearman’s *r*=−0.43, *p* = 0.0007) were negatively correlated with relapse frequency (**Figures 6F, G**; **Supplementary Figure 8**). Thus, immune cell cytokine expression patterns allowed patients to be stratified by relapse frequency independently of conventional clinical indicators, suggesting potential for immune cell-based signatures to serve as biomarkers for disease monitoring and assessing relapse risk in rGPA patients.

## Discussion

Current treatments for GPA have improved survival of affected individuals; however, despite these advances, disease relapse in patients who have achieved remission remains a clinical challenge. Prolonging immunosuppressive therapy may reduce relapse likelihood, but is associated with infections and other risks and remains of questionable clinical benefit. There is, therefore, a significant need for novel approaches to extend remission and, by extension, for biomarkers that enable prediction and prevention of disease relapse.

Changes in ANCA positivity and/or levels as well as numbers of specific B cell subsets have been considered as possible predictors of relapse risk in GPA (20, 22). However, currently the reliability and sensitivity of these alterations in predicting disease flare remain unclear. While these studies have largely focused on correlations of relapse risk with single marker or cell subsets, we leveraged high-dimensional mass cytometry to capture a more comprehensive picture of the immunophenotypic profile associated with GPA remission. Furthermore, we utilized ML methods to analyze these data and develop a model for detecting individuals with high likelihood for disease relapse.

We observed significant reductions in numbers and frequencies of circulating T lymphocytes and most T cell subsets in rGPA patients compared with HCs. Moreover, both CD4^+^ and CD8^+^ T cells from patients were shifted toward an effector/memory phenotype, suggesting a relative expansion of antigen-driven T cell activation despite the absence of overt disease activity. Reduced numbers and functional impairment of T regulatory cells have been reported in association with active disease in GPA, but in contrast to the rGPA patients studied here, active GPA appears to be associated with enhanced activation of effector T cells that drives increased pro-inflammatory responses. However, among the rGPA patients studied here, effector T cells exhibited reduced expression of activation markers (CD69) and pro-inflammatory cytokines (TNF-α, IFN-γ, IL-2, IL-17α), implying a state of immune exhaustion or anergy rather than sustained hyperactivation. Similarly, Vδ2^+^ γδ T cells exhibited features of terminal exhaustion (reduced CD56, increased PD-1), suggesting impairment of γδ T cell functions. Thus, while the use of immunosuppressive medications by the majority of rGPA patients studied likely contributes to their T cell status, the T cell immune responses associated with clinical remission in these patients significantly differ from those observed in HCs.

As observed for T lymphocytes, numbers of total circulating B cells and all B cell subsets were significantly reduced in rGPA patients compared with HCs. However, despite their reduced numbers, B cells from the rGPA group exhibited features consistent with increased activation, including reduced naïve B cell and increased memory B cell frequencies, and elevated expression of pro-inflammatory cytokines including IL-6 and IL-17α. Thus, despite the use of B cell depleting therapies by many rGPA patients, persisting increased B cell activation may represent a factor influencing likelihood of relapse.

Immune profiling of rGPA patients also revealed sustained activation of innate immune subsets, particularly CD123^+^ monocytes and activated DCs, suggesting inflammatory/proinflammatory state. Sustained expansion of inflammatory monocyte subsets in rGPA patients was associated with elevated production of IL-6, TNF-α, and IL-8, implicating these cells in driving persistent innate immune activation during remission. Similarly, the increased frequency of CD177^+^ neutrophils in rGPA patients is noteworthy given the essential role for CD177 in promoting PR3 presentation on the neutrophil surface and consequent ANCA-mediated neutrophil activation and neutrophil-driven tissue injury.^39^ The specific expansion of CD177^+^ neutrophils expressing CD11b and CD49d, markers of neutrophil activation and migratory potential, respectively, further reinforces the persistence of hyperactivated innate immune responses despite remission status of the GPA patients. Similarly, increases in activated CD56^dim^ NK cells with upregulated expression of CD69 and TNFα in the rGPA patients suggest a shift toward a pro-inflammatory phenotype, consistent with an immune response poised for perpetuating inflammation and, by extension, potential for relapse.

In addition to characterizing immune dysregulation associated with rGPA, this study leveraged machine-learning techniques to identify four immune biomarkers—γδ T cell depletion, CD177^+^ neutrophil expansion, monocyte expansion, and B cell depletion—that show high accuracy in partitioning rGPA patients from HCs. An ML model also delineated three distinct clusters of immunophenotypic profiles in this patient population, including one cluster characterized by heightened inflammatory cytokine expression and enrichment for rGPA patients with histories of frequent relapses. A risk score based on this analysis, and predicated on altered levels of IL-8 in monocytes and of IL-10 in monocytes and cDC2s cells, was highly associated with increased relapse frequency in rGPA patients. These findings reveal the potential for immune phenotyping to identify markers associated with disease/disease outcomes and for ML methods to inform the interpretation and clinical utility of such data (44–46).

Another immune profiling study on GPA patients has also highlighted the occurrence of monocyte alterations in GPA (47) (*Bonasia* et al.*, 2024*). This study reported that monocyte frequencies were increased in patients with active GPA compared to the frequencies observed in remission patients and healthy controls. By contrast, our study has focused on immune profiles from rGPA patients, revealed the frequency of monocytes to be increased and their cytokine profiles to be altered in these patients and linked these changes with relapse frequency. This finding is consistent with results of an earlier study showing that the frequency of activated monocytes was increased in MPA patients both before and after immunosuppressive therapy and was associated with a higher rate of relapse (48) (*Nishide* et al.*, 2023*). In addition, while increased T-cell activation is well documented in active GPA, our findings reveal that aberrant T-cell activation persists in rGPA. Collectively, our data suggest that dysregulation of both monocytes and T cells may increase the risk of GPA relapse.

Despite the encouraging results, our study has some limitations. First, the study subjects were all recruited from a single center and constituted a relatively small cohort or primarily PR3-ANCA positive patients, factors that may limit the generalizability of the conclusions. In addition, almost all of the rGPA patients were receiving remission maintenance therapy (corticosteroids and/or immunosuppressive medications), potentially confounding the interpretation of the immune cell alteration profiles. Lastly, the models were developed and validated using internal cross-validation and a random train-test split, rather than an independently ascertained validation cohort. Future multi-center studies incorporating larger and more diverse patient populations including those affected by MPA, longitudinal sampling, and independent external validation are required to validate these findings and evaluate their potential clinical applicability.

Our study provides novel insights into the immune dysregulation associated with GPA in clinical remission, identifying ongoing suppressed T cell responses and heightened B and myeloid cell activation in these patients and revealing the potential for ML models trained on complex immunophenotypic data to inform development of clinically predictive risk scores for guiding therapeutic decisions.

## Data Availability

The original contributions presented in the study are included in the article/**Supplementary Material**. Further inquiries can be directed to the corresponding author.
