# Correction: Comprehensive immune profiling identifies alterations in adaptive and innate immune responses in granulomatosis with polyangiitis patients in remission

**DOI:** 10.3389/fimmu.2026.1874627

**Published:** 2026-05-13

**Authors:** Ming Liu, Deming Duan, Chenghao Li, Xia Hu, Jinghao Huang, Yue Tang, Steven Palayew, Lei Zhang, Christian Pagnoux, Cynthia Guidos, Jinyi Zhang, Katherine Siminovitch

**Affiliations:** 1 Mount Sinai Hospital, Lunenfeld-Tanenbaum and Toronto General Hospital Research Institutes, Toronto, ON, Canada; 2 Oujiang Laboratory (Zhejiang Lab for Regenerative Medicine, Vision and Brain Health), Wenzhou Medical University, Wenzhou, Zhejiang, China; 3 School of Life Science and Technology, State Key Laboratory of Urban Water Resource and Environment, Harbin Institute of Technology, Harbin, Heilongjiang, China; 4 College of Basic Medicine, Jinzhou Medical University, Jinzhou, Liaoning, China; 5 Vasculitis Clinic, Division of Rheumatology, Mount Sinai Hospital, University of Toronto, Toronto, ON, Canada; 6 The Hospital for Sick Children Research Institute, Program in Cell & Systems Biology, University of Toronto, Toronto, ON, Canada; 7 Department of Immunology, Faculty of Medicine, University of Toronto, Toronto, ON, Canada; 8 Department of Medicine, Faculty of Medicine, University of Toronto, Toronto, ON, Canada

**Keywords:** granulomatosis with polyangiitis, immune dysregulation, immunophenotypes, innate immunity, lymphocytes, machine learning, mass cytometry, precision medicine

The funder “National Natural Science Foundation of China, (32300748)” was erroneously omitted. The corrected **Funding** statement appears below:

***“***The author(s) declared that financial support was received for this work and/or its publication. This work was supported by the Erna Baird Memorial Grant, the Vasculitis Foundation of Canada, the Ontario Research Fund (grant RE-09-090), the Canadian Institutes of Health Research (PJT186044), the Sherman Family Chair in Genomic Medicine, and the National Natural Science Foundation of China (32270940, 32300748). CyTOF was performed at the SickKids’ Centre for Advanced Single Cell Analysis and supported by the SickKids Foundation and a Canadian Foundation for Innovation Fund John Evans Fund Leaders’ Infrastructure Grant to CG (#37562). CG’s work was supported by a Canadian Institutes of Health Research Project Grant (FRN 165973).”

