# Supplementary material for: Comprehensive immune profiling identifies alterations in adaptive and innate immune responses in granulomatosis with polyangiitis patients in remission
Source: Front Immunol. 2026 Mar 27;17:1726107. doi: 10.3389/fimmu.2026.1726107 (PMC13066301; doi:10.3389/fimmu.2026.1726107)
Supplement: Supplementary file 1 [file DataSheet1.pdf]

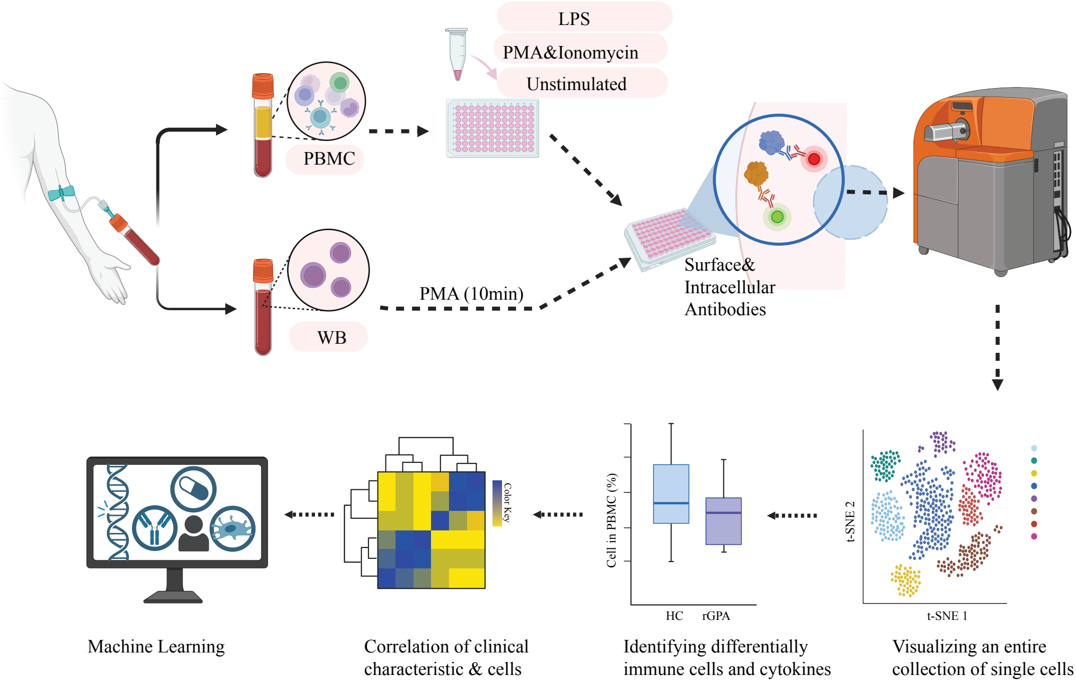

Supplementary Figure 1. Study design and analytical pipeline. Whole blood (WB) from HCs and rGPA patients was collected, and PBMCs were isolated using SepMate™ and LymphPrep. WB and PBMCs were separately stimulated with PMA, PMA/Ionomycin, LPS, or left unstimulated, followed by antibody staining and mass cytometry analysis. Data were analyzed in Cytobank. viSNE was used for high-dimensional visualization to identify differentially abundant immune cell subsets and manual gating used to quantify adaptive and innate populations. Machine learning models were trained on diagnostic samples to identify immune cell features distinguishing rGPA from HCs and rGPA patients with increased likelihood of relapse.
