# Supplementary material for: Comprehensive immune profiling identifies alterations in adaptive and innate immune responses in granulomatosis with polyangiitis patients in remission
Source: Front Immunol. 2026 Mar 27;17:1726107. doi: 10.3389/fimmu.2026.1726107 (PMC13066301; doi:10.3389/fimmu.2026.1726107)
Supplement: Supplementary file 2 [file DataSheet2.pdf]

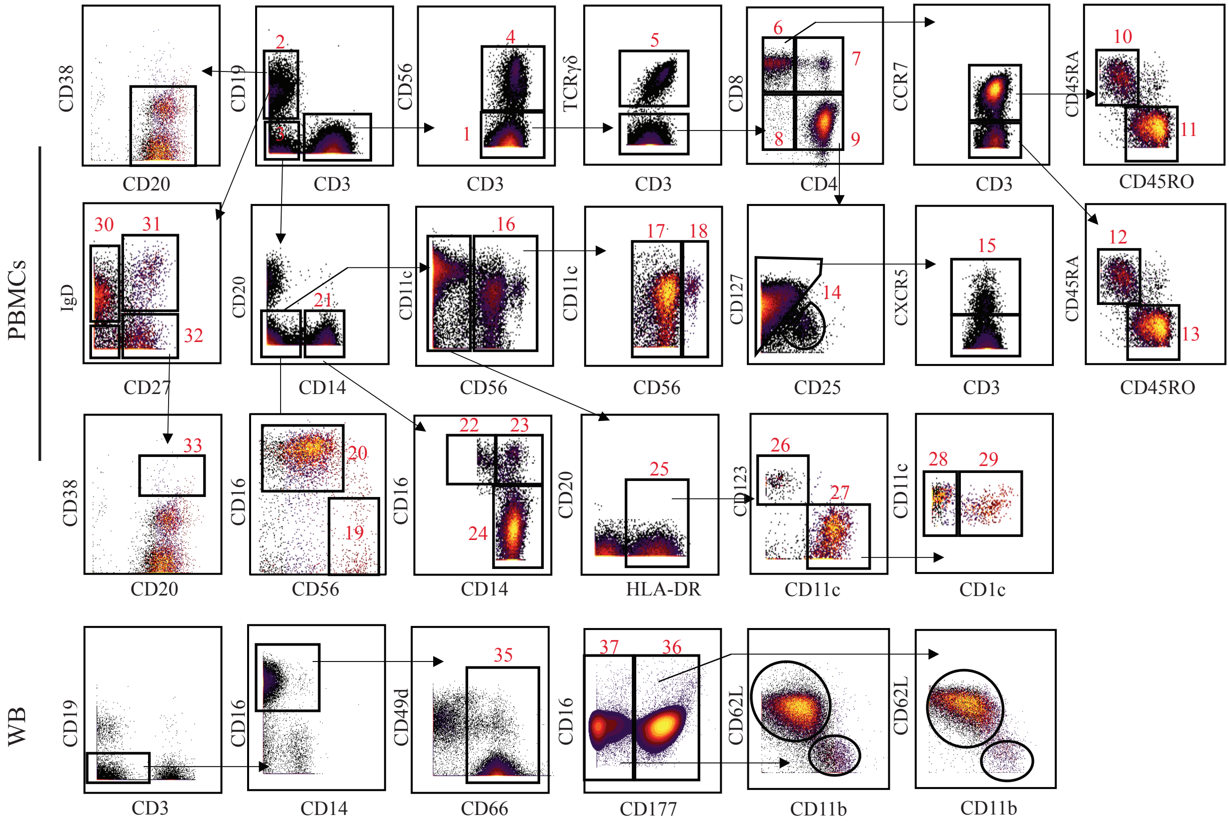

Supplementary Figure 2. Gating strategy for immune cell subsets in PBMCs and WB. Representative two-dimensional scatter plots show the sequential manual gating strategy performed in Cytobank. Both adaptive and innate immune cell subsets were identified. Subset numbering corresponds to the definitions provided in Supplementary Table 3.
