# Supplementary material for: Comprehensive immune profiling identifies alterations in adaptive and innate immune responses in granulomatosis with polyangiitis patients in remission
Source: Front Immunol. 2026 Mar 27;17:1726107. doi: 10.3389/fimmu.2026.1726107 (PMC13066301; doi:10.3389/fimmu.2026.1726107)
Supplement: Supplementary file 3 [file DataSheet3.pdf]

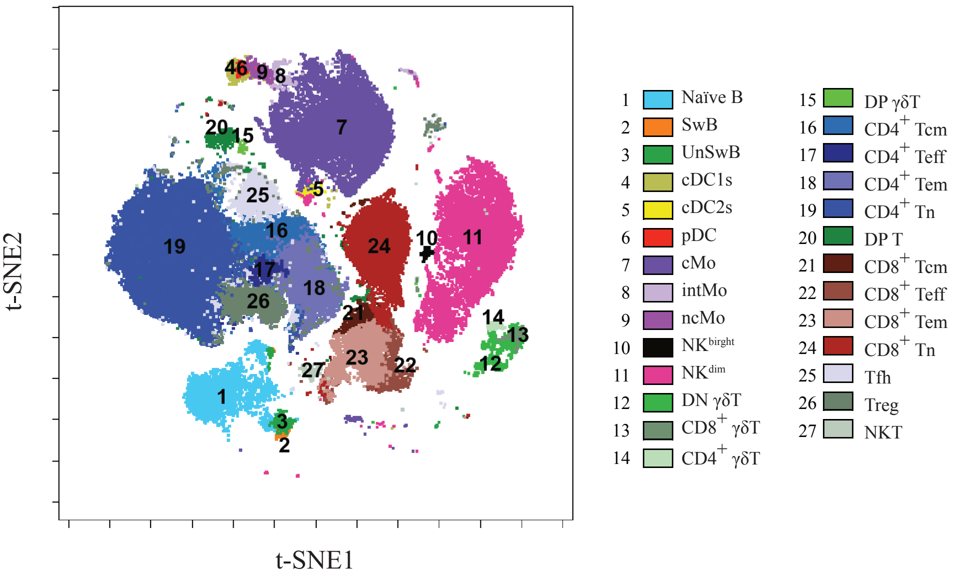

Supplementary Figure 3. viSNE plots of all CyTOF samples. viSNE visualization of the phenotyping panel dataset including 31 HCs and 59 rGPA blood samples, with 2,000 randomly selected cells per sample. Each point represents a single cell, colour-coded according to merged and annotated clusters. This visualization enables comparison of immune cell composition patterns across groups.
