# Supplementary material for: Comprehensive immune profiling identifies alterations in adaptive and innate immune responses in granulomatosis with polyangiitis patients in remission
Source: Front Immunol. 2026 Mar 27;17:1726107. doi: 10.3389/fimmu.2026.1726107 (PMC13066301; doi:10.3389/fimmu.2026.1726107)
Supplement: Supplementary file 4 [file DataSheet4.pdf]

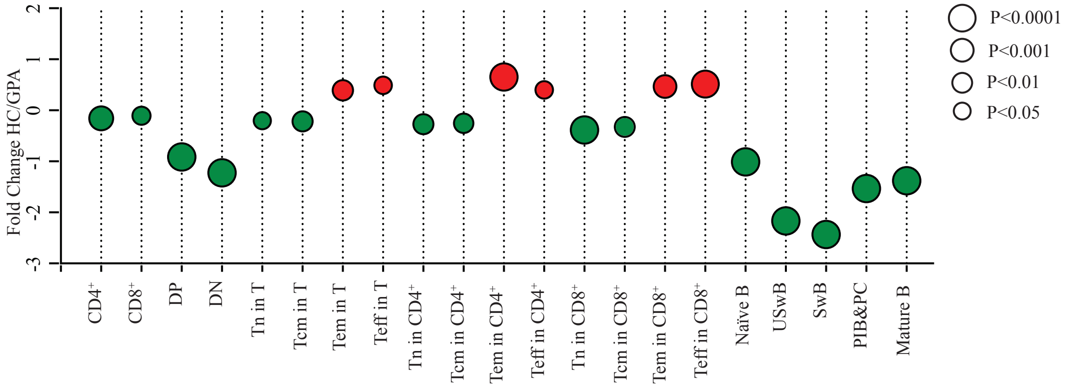

Supplementary Figure 4. Fold changes and significance of T and B cell subsets in PBMCs. Bubble chart showing fold changes and statistical significance of T and B cells and their subpopulations between rGPA and HCs samples. Bubble size indicates significance level; green bubbles indicate decreased abundance, and red bubbles indicate increased abundance in rGPA.
