# Supplementary material for: Comprehensive immune profiling identifies alterations in adaptive and innate immune responses in granulomatosis with polyangiitis patients in remission
Source: Front Immunol. 2026 Mar 27;17:1726107. doi: 10.3389/fimmu.2026.1726107 (PMC13066301; doi:10.3389/fimmu.2026.1726107)
Supplement: Supplementary file 5 [file DataSheet5.pdf]

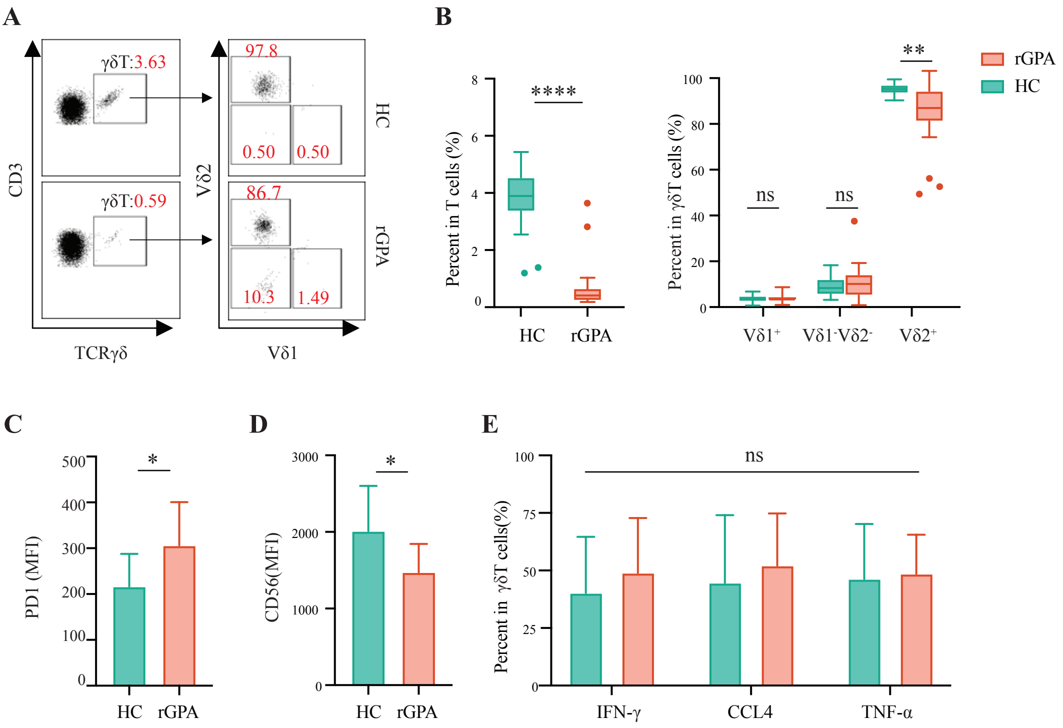

Supplementary Figure 5. γδT cells are depleted in rGPA patients. PBMCs from HCs and rGPA patients were either untreated or stimulated with 50 ng/ml PMA plus 500 ng/ml ionomycin. (A) Representative flow cytometry plots showing γδT cells (left) and $V\delta1^+$, $V\delta1^-V\delta2^-$ and $V\delta2^+$ subsets (right) within γδT cell population in PBMCs from HCs and rGPA patients. (B) Frequencies of γδT cells in $CD3^+$T cells (left) and $V\delta1^+$, $V\delta1^-V\delta2^-$ and $V\delta2^+$ subsets within γδT cells (right) in PBMCs from HCs and rGPA patients. Quantified expression of PD1 (C) and CD56 (D) on $V\delta2^+$ cells, measured by mean fluorescence intensity (MFI). (E) Frequency of IFN-γ (left), CCL4 (middle), and TNF-α (right), producing $V\delta2^+$ cells following PMA/Ionomycin stimulation in HCs and rGPA patients. N=15 for HCs and 19 for rGPA samples. Data are presented as Tukey-style box-and-whisker plots (B). Center line: median; box: first/third quartiles; whiskers: 1.5xIQR; outliers shown. Or presented as bar graphs with mean ± SD [(C) to (E)]. Statistical significance was assessed using two-sided Mann-Whitney U test, and false discovery rate (FDR) was controlled at 5% using the Benjamini–Hochberg procedure. *$p<0.05$, **$p<0.01$, ****$p<0.0001$; ns: not significant.
