# Supplementary material for: Comprehensive immune profiling identifies alterations in adaptive and innate immune responses in granulomatosis with polyangiitis patients in remission
Source: Front Immunol. 2026 Mar 27;17:1726107. doi: 10.3389/fimmu.2026.1726107 (PMC13066301; doi:10.3389/fimmu.2026.1726107)
Supplement: Supplementary file 6 [file DataSheet6.pdf]

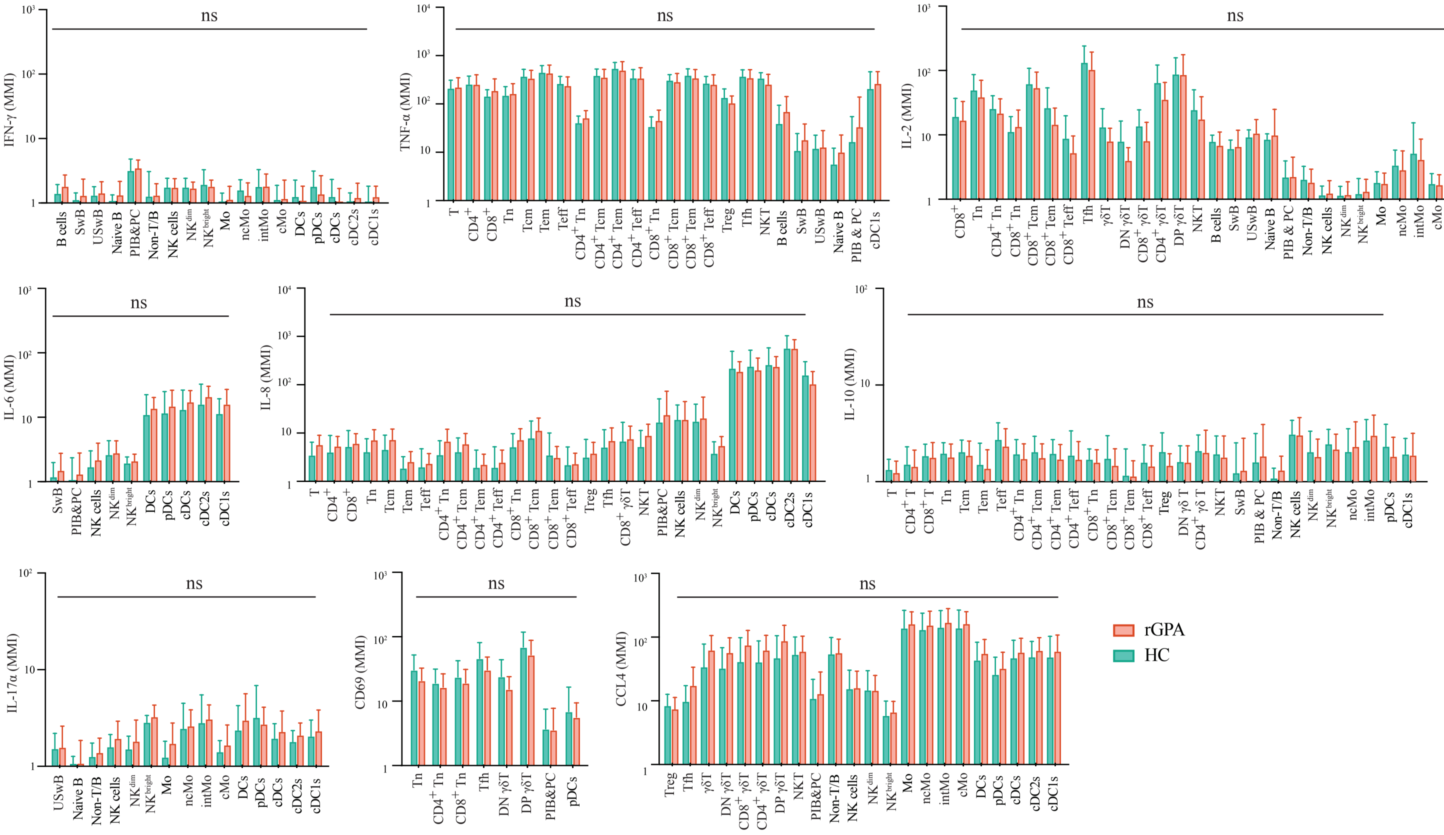

Supplementary Figure 6. No significant expression of inflammatory cytokines in adoptive and innate cell subsets in PBMCs from rGPA patients. PMA/Ionomycin- or LPS-induced IL-2, IFN-γ, TNF-α, IL-17α, CD69, IL-8, CCL4, IL-10 and IL-6 expression in indicated cell subsets in PBMCs from HCs and rGPA patients. Data are shown as mean ± SD. n=31 for HCs and n=59 for rGPA. Statistical analysis was performed using two-sided Mann-Whitney U test. ns: not significant.
