# Supplementary material for: Comprehensive immune profiling identifies alterations in adaptive and innate immune responses in granulomatosis with polyangiitis patients in remission
Source: Front Immunol. 2026 Mar 27;17:1726107. doi: 10.3389/fimmu.2026.1726107 (PMC13066301; doi:10.3389/fimmu.2026.1726107)
Supplement: Supplementary file 7 [file DataSheet7.pdf]

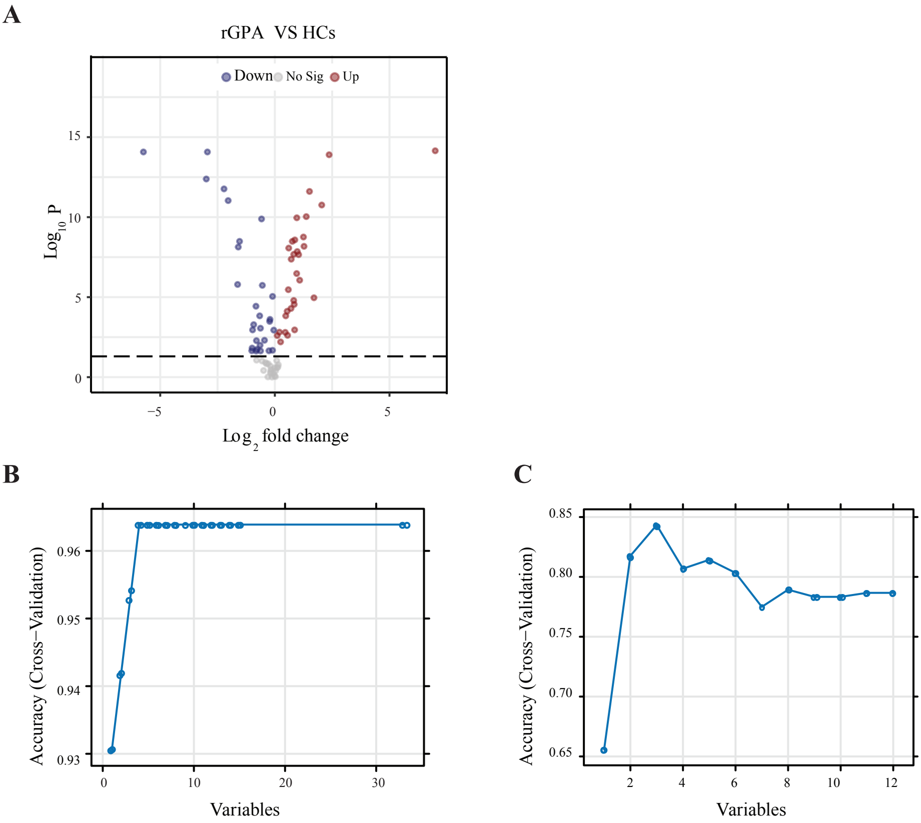


**Supplementary Figure 7. Significant features.** (A) Volcano plot of differential immune cell subset frequencies in rGPA versus HCs. X-axis: $\log_2$ fold change (rGPA/HCs); y-axis: $\log_{10}$ p-value from two-sided Wilcoxon rank-sum test. Red: significantly increased ($p<0.05$; n=30); blue: significantly decreased ($p<0.05$; n=29); grey: non-significant ($p\geq0.05$). Horizontal dashed line: $p=0.05$ ($\log_{10}=1.3$); vertical dashed lines: $\log_2$ fold-change ± 1. (B) Recursive feature elimination (RFE) process for selecting biomarkers to distinguish rGPA from HC in the diagnostic model. (C) RFE results showing selected predictors for distinguishing relapsing from non-relapsing rGPA patients.
