# Supplementary material for: Comprehensive immune profiling identifies alterations in adaptive and innate immune responses in granulomatosis with polyangiitis patients in remission
Source: Front Immunol. 2026 Mar 27;17:1726107. doi: 10.3389/fimmu.2026.1726107 (PMC13066301; doi:10.3389/fimmu.2026.1726107)
Supplement: Supplementary file 8 [file DataSheet8.pdf]

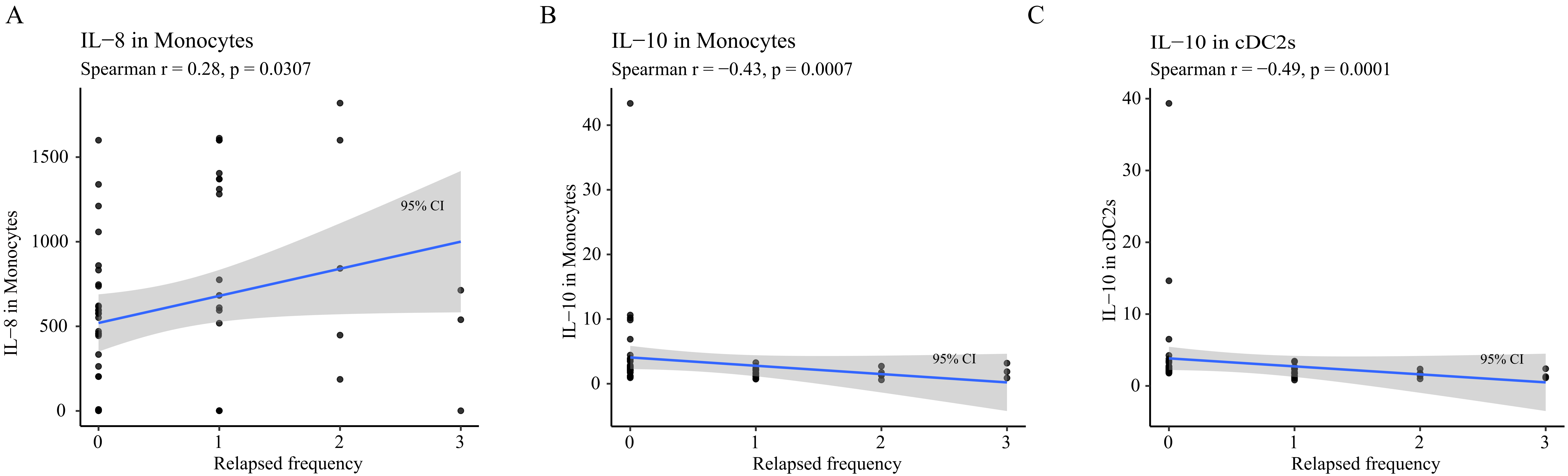

Supplementary Figure 8. Linear regression fits for relapse frequency associated cytokine features. Scatter plots show the relationship between relapse frequency and cytokine expression levels in (A) IL-8 in monocytes, (B) IL-10 in monocytes, and (C) IL-10 in cDC2s. Each dot represents one patient. Blue line: fitted linear regression (lm) trend; grey shaded band: 95% confidence interval. Spearman correlation coefficients and p values are indicated in each panel
