# Supplementary material for: Comprehensive immune profiling identifies alterations in adaptive and innate immune responses in granulomatosis with polyangiitis patients in remission
Source: Front Immunol. 2026 Mar 27;17:1726107. doi: 10.3389/fimmu.2026.1726107 (PMC13066301; doi:10.3389/fimmu.2026.1726107)
Supplement: Supplementary file 9 [file Supplementaryfile1.docx]

Supplementary Material

**Supplementary Materials and Methods**

***Blood Sampling and Processing***

Peripheral venous WB from rGPA patients and HCs was collected in three Vacutainer Heparin tubes (BD Bioscience, #367874) kept at room temperature and processed immediately after collection. Fresh WB was stained immediately with a 2× concentration of a surface antibody cocktail, along with Human TruStain FcX^TM^ (Biolegend, #422302) for Fc receptor blocking, for a total of 30 min at room temperature (RT). During the final 10 minutes, phorbol 12-myristate 13-acetate (PMA; 500 ng/mL) was added, and the samples were incubated at 37°C, with non-PMA treated control tubes prepared in parallel. Following staining and stimulation, red blood cells were lysed using BD Pharm Lyse™ Lysing Buffer (#555899) for 15 minutes at RT. The cells were then washed twice with PBS to remove residue serum or lysis buffer and stained for 5 minutes at RT with 2 μM cisplatin (Bio Vision, #1550-1000) for viability assessment, followed by quenching with 5% heat-inactivated Fetal Bovine Serum (FBS) in PBS. Samples were subsequently fixed and permeabilized with 1.6% paraformaldehyde (PFA) in PBS containing 0.3% saponin for 15 minutes at RT in the dark. Following fixation, cells were incubated overnight at 4°C with 100 nM iridium intercalator (Fluidigm, #201192B) diluted in PBS containing 0.3% saponin for DNA content labeling. The following morning, samples were washed with PBS, passed through a 35-μm strainer (Falcon, #352235), and resuspended in PBS.

In parallel with WB processing, peripheral blood mononuclear cells (PBMCs) were obtained by density gradient centrifugation using SepMate™ tubes (StemCell Inc., #85460) and LymphPrep (density 1.077 g/ml; StemCell Inc., #07861) according to manufacturer’s instructions. Cells were then washed twice with PBS containing 1% Bovine Serum Albumin, counted, pelleted and resuspended at 5 × 10^6^ cells per vial in freezing medium (90% heat-inactivated FBS + 10% DMSO). Controlled-rate freezing was performed using a Mr. Frosty container (Thermo Fisher Scientific, #5100-0001) at approximately -1°C/min, and vials were then stored in liquid nitrogen.

Frozen PBMCs from rGPA patients and HCs were thawed in a 37°C water-bath for 1–2 minutes without agitation until only a small ice crystal remained. Cells were then slowly mixed with pre-warmed culture media (IDMD supplemented with 10% heat-inactivated FBS, 25 mM HEPES, 0.1 mM non-essential amino acid, 1 mM sodium pyruvate, 2 mM L-glutamine, 100 U/ml penicillin-streptomycin and 55 μM 2-mercaptoethanol; from Gibco or Thermo Fisher Scientific) and pelleted at 300 g for 8 min at RT, then resuspended in 10 ml of pre-warmed culture media and recovered for 30 minutes in 37°C with 5% CO₂. Cell debris was removed using 70-μm cell strainer (Falcon, #352350). For stimulation, PBMCs were treated with PMA (50 ng/ml) and ionomycin (500 ng/ml) for T cell lineages, or with LPS (1 μg/ml) for B cells and innate immune cells, for a total of 5 hours at 37°C with 5% CO₂. Brefeldin A (1:1000; Biolegend, #420601) and monensin (1:1000; Biolegend, #420701) were added during the last 4 hours of stimulation. After stimulation, Fc receptors were blocked for 10 minutes at RT, and PBMC were stained for 30 minutes at RT with a 2× concentration of a surface antibody cocktail targeting PBMC (PBMC panel; **Supplementary Table 1**). Cells were then washed and stained with cisplatin, then fixed and permeabilized. Intracellular staining was performed using a PBMC intracellular antibody cocktail (PBMC panel; **Supplementary Table 1**), followed by incubation with 100 nM iridium intercalator for DNA content labeling. The next morning, samples were washed, filtered, resuspended with PBS and submitted for mass cytometry analysis.

***Mass Cytometry Staining and Acquisition***

The antibody panels, stimulation conditions, and intracellular staining protocols used for mass cytometry in this study were first optimized and validated by flow cytometry on a BD Fortessa X20. CD45-89Y was purchased from Standard BioTools. All other antibodies were purchased from Biolegend and conjugated with indicated metal isotopes using Maxpar X8 Antibody Labeling Kits (Standard BioTools) by the Centre for Advanced Single Cell Analysis (CASCA) at the Hospital for Sick Children (Toronto, Ontario, Canada) following the manufacturer’s protocol. Details of the antibody panels, including mass tags, target antigens, clones, and associated subpopulations, are provided in **Supplementary Table 1**. Titrations for newly acquired or new lots of antibodies were performed at dilutions of 1:90, 1:270, 1:810, and 1:2430. Optimal dilutions were determined based on signal quality assessed by mass cytometry analysis and subsequently applied for antibody staining. Before staining, antibody cocktails were filtered through a 0.1-μm Ultrafree® 500 μL-MC filter (Millipore, #FC30VV00) and centrifuged at 13,000 × g for 3 minutes at 4°C. The clarified antibody solutions were then used for staining.

The stained samples were analyzed on a Helios mass cytometer (Standard Biotools) equipped with a wide-bore injector according to the manufacturer’s protocols as previously described (49, 50). Acquisition was performed at a rate of 100–250 events per second, with 100,000 events collected per sample. The Helios software (v6.7.1014) was used to normalize and then de-barcode with the CyTOF Software v6 debarcoder, and then uploaded to CytoBank (Beckman Coulter Enterprise license) where manual pre-gating was performed to remove debris, dead cells, doublets.

***Flow cytometry analysis of γδ T cell subsets***

For γδ T cell subsets phenotyping, we performed a focused follow-up analysis after discovery analyses indicated a reduction of γδ T cells in rGPA patients. Inclusion criteria were availability of residue PBMCs and post-thaw viability greater than 80%. Briefly, HC or rGPA PBMCs were thawed and stimulated as described above. PBMCs were then subjected to stain with fluorophor-conjugated anti-CD3 (clone UCHT1), anti-TCR-γδ (clone 5A6.E9), anti-Vδ1 (clone R9.12), anti-Vδ2 (clone 123R3), anti-PD1 (clone EH12.2H7) and anti-CD56 (clone NCAM16.2) antibodies, and followed by staining with anti-IFN-γ (clone B27), anti-CCL4 (clone D21-1351) and anti-TNF-α (clone MAb11) antibodies. Data were acquired on BD Fortessa and analyzed by FlowJo.

***Construction of the Machine Learning Models***

Identification of Candidate Features: We used the Wilcoxon rank-sum test to assess differences in immune cell subset abundance between HCs and rGPA patients, and also between relapsed and non-relapsed rGPA patients. We used the same test to evaluate cytokine levels between relapsed and non-relapsed rGPA patients. Next, pairwise Pearson correlation coefficients were calculated to address potential multicollinearity among candidate features. For any pair of features with an absolute correlation coefficient greater than 0.7 (|r| > 0.7), the feature with lower initial importance—based on preliminary random forest rankings—was removed. This filtering step reduced redundancy and improved model interpretability and stability.

Recursive Feature Elimination (RFE): Feature selection was implemented using a random forest-based recursive feature elimination (RF-RFE) approach. The *rfe* function in caret with random forest-based ranking (rfFuncs) was applied to iteratively rank features by importance and eliminate the least informative variables. Ten-fold cross-validation repeated five times (*method = "repeatedcv"*) was used to optimize feature subset selection. The final optimal subset was determined based on cross-validated model performance, and selected features were extracted using the *predictors* function. The relationship between the number of features and model performance was visualized to guide final selection.

Model Development and Hyperparameter Tuning: Final model training was conducted using the train function in caret, with class probability estimation enabled (*classProbs = TRUE*) and stratified upsampling (*sampling = "up"*) to address class imbalance. Hyperparameter tuning tested *mtry* values of 2, 5, and 10, while the number of trees was fixed at 100 (*ntree = 100*). Model performance was optimized using the twoClassSummary metric during training. The model training was performed based on randomly 80% of total samples, while the remaining 20% were held out as an independent internal test set to evaluate the performance.

Performance Evaluation and Classification Threshold: Model discrimination was primarily assessed using receiver operating characteristic (ROC) curves, and the area under the ROC curve (AUC) was calculated to quantify predictive power. A fixed classification threshold of 0.5 was applied: MICG (Multi-Immune Cell Feature GPA) or MICGR (Multi-Immune Cell Feature GPA Relapse) scores ≥ 0.5 were classified as GPA or relapsed GPA, respectively, while scores < 0.5 were considered HCs or non-relapsed GPA. To further validate model performance at this threshold, additional metrics including overall accuracy, positive predictive value (PPV), and negative predictive value (NPV) were also calculated and reported.

**Supplementary Figure Legends**

**Supplementary Figure 1. Study design and analytical pipeline.** Whole blood (WB) from HCs and rGPA patients was collected, and PBMCs were isolated using SepMate™ and LymphPrep. WB and PBMCs were separately stimulated with PMA, PMA/Ionomycin, LPS, or left unstimulated, followed by antibody staining and mass cytometry analysis. Data were analyzed in Cytobank. viSNE was used for high-dimensional visualization to identify differentially abundant immune cell subsets and manual gating used to quantify adaptive and innate populations. Machine learning models were trained on diagnostic samples to identify immune cell features distinguishing rGPA from HCs and rGPA patients with increased likelihood of relapse.

**Supplementary Figure 2. Gating strategy for immune cell subsets in PBMCs and WB.** Representative two-dimensional scatter plots show the sequential manual gating strategy performed in Cytobank. Both adaptive and innate immune cell subsets were identified. Subset numbering corresponds to the definitions provided in Supplementary Table 3.

**Supplementary Figure 3. viSNE plots of all CyTOF samples.** viSNE visualization of the phenotyping panel dataset including 31 HC and 59 rGPA blood samples, with 2,000 randomly selected cells per sample. Each point represents a single cell, colour-coded according to merged and annotated clusters. This visualization enables comparison of immune cell composition patterns across groups.

**Supplementary Figure 4. Fold changes and significance of T and B cell subsets in PBMCs.** Bubble chart showing fold changes and statistical significance of T and B cells and their subpopulations between 31 HC and 59 rGPA samples. Bubble size indicates significance level; green bubbles indicate decreased abundance, and red bubbles indicate increased abundance in rGPA.

**Supplementary Figure 5. γδ T cells are depleted in rGPA patients.** PBMCs from HCs and rGPA patients were either untreated or stimulated with 50 ng/ml PMA plus 500 ng/ml ionomycin. (A) Representative flow cytometry plots showing γδ T cells (*left*) and Vδ1^+^, Vδ1^-^Vδ2^-^ and Vδ2^+^ subsets (*right*) within γδ T cell population in PBMCs from HCs and rGPA patients. (B) Frequencies of γδ T cells in CD3^+^T cells (*left*) and Vδ1^+^, Vδ1^-^Vδ2^-^ and Vδ2^+^ subsets within γδ T cells (*right*) in PBMCs from HCs and rGPA patients. Quantified expression of PD1 (C) and CD56 (D) on Vδ2^+^ cells, measured by mean fluorescence intensity (MFI). (E) Frequency of IFN-γ (*left*), CCL4 (*middle*), and TNF-α (*right*), producing Vδ2^+^ cells following PMA/Ionomycin stimulation in HCs and rGPA patients. N=15 for HCs and 19 for rGPA samples. Data are presented as Tukey-style box-and-whisker plots (B). Center line: median; box: first/third quartiles; whiskers: 1.5xIQR; outliers shown. Or presented as bar graphs with mean ± SD [(C) to (E)]. Statistical significance was assessed using two-sided Mann-Whitney U test. **p*<0.05, ***p*<0.01, *****p*<0.0001; ns: not significant.

**Supplementary Figure 6. No significant expression of inflammatory cytokines in adoptive and innate cell subsets in PBMCs from rGPA patients.** PMA/Ionomycin- or LPS-induced IL-2, IFN-γ, TNF-α, IL-17α, CD69, IL-8, CCL4, IL-10 and IL-6 expression in indicated cell subsets in PBMCs from 31 HCs and 59 rGPA patients. Data are shown as mean ± SD. Statistical analysis was performed using two-sided Mann-Whitney U test. ns: not significant.

**Supplementary Figure 7. Significant features.** (A) Volcano plot of differential immune cell subset frequencies in rGPA versus HCs. X-axis: log_2_ fold change (rGPA/HCs); y-axis: log_10_ *p*-value from two-sided Wilcoxon rank-sum test. Red: significantly increased (p<0.05; n=30); blue: significantly decreased (p<0.05; n=29); grey: non-significant (p≥0.05). Horizontal dashed line: *p*=0.05 (log_10_=1.3); vertical dashed lines: log_2_ fold-change ± 1. (B) Recursive feature elimination (RFE) process for selecting biomarkers to distinguish rGPA from HCs in the diagnostic model. (C) RFE results showing selected predictors for distinguishing relapsing from non-relapsing rGPA patients.

**Supplementary Table 2**. Antibody panels for CyTOF-based immunophenotyping.

**Supplementary Table 3**. Patient characteristics at study inclusion.

**Supplementary Table 4**. Abbreviations of immune cell subsets and the gating strategy applied in Cytobank for their identification.
